# Confounding factors affecting the clinical decision-making of nursing and midwifery students post-pandemic COVID-19: cross-sectional study in Jordan

**DOI:** 10.1186/s12912-024-02108-3

**Published:** 2024-06-24

**Authors:** Rafi Alnjadat, Eshraq Almomani, Lourance Al Hadid, Amer Al-Omari, Alaa Fraihat

**Affiliations:** 1 Irbid University College, Al-Balqa Applied University, P.O. Box: 20, Irbid, 22110 Jordan; 2 Faculty of Nursing, Al-Balqa Applied University, Irbid, Jordan; 3 Applied Science Departmnet, Ajloun University College, Al-Balqa Applied University, Ajloun, Jordan

**Keywords:** Nursing students, Clinical decision making, Social media, Confounding factor

## Abstract

**Background:**

The ability of a nurse to make effective clinical decisions is the most important factor that can affect the treatment quality. However, several factors can affect the ability of nursing and midwifery students to make effective clinical decisions.

**Objectives:**

This study aims to identify the confounding factors that may affect the clinical decision making of nurses and thus patient outcomes after the COVID-19 pandemic in Jordan.

**Methods:**

A descriptive cross-sectional design was employed in this study. An online self-administered questionnaire was distributed to 269 nursing and midwifery students selected through purposive sampling, 224 of whom completed the questionnaire. The valid and reliable nursing decision-making instrument, which consisted of 24 items, was employed to gather the data, and descriptive statistics and simple linear regression were employed for the data analysis. Data was collected from November to the end of December 2022.

**Results:**

Among the respondents, 72.8% were female, and the average age was 20.79 years (SD = 1.44). The vast majority of the respondents (94.6%) was unmarried, and 74.1% were pursuing a nursing degree. The simple linear regression analysis showed that clinical decision making had a negative and significant relationship with social media usage of an average of 6 h a day (β=−0.085). Moreover, the male nursing students obtained lower clinical decision-making scores (β= −0.408) compared with the female nursing students.

**Conclusion:**

Social media usage and gender have a considerable effect on the clinical decision making of the nursing and midwifery students. Therefore, the confounding factors that can affect the clinical decision making of nurses should be discussed further, and strategies to address such factors should be implemented.

## Introduction

The COVID-19 pandemic affected economies, societies, and governments and had a more severe impact than previous recessions. Specifically, the lockdowns caused businesses to close, disrupted supplies, and forced the general population to stay at home. The COVID-19 pandemic occurred in the present era, when a successful manager is believed to have a strategic mind [1].

Several factors influenced how the European higher education sector responded to various pandemic-related crises, which also affected the sector’s post-pandemic image in terms of internationalization. The pandemic had a considerable impact on several aspects of society, including mobility, domestic internationalization, and regional social responsibility agendas. Such issues will continue to be important in the post-pandemic future [2].

The life-threatening situations brought on by COVID-19 caused medical education to face difficulties, such as in maintaining its integrity and consistency while ensuring that faculty members could deliver lectures safely [3]^,^ As a result of such difficulties, patient treatment was constrained, as focus shifted to confirm COVID-19 cases, which restricted medical students’ access to bedside education [4]. In addition, clinical rotations were postponed, because medical students could contract the virus during their training and subsequently infect the local population [5, 6]. Therefore, students were instructed to stay at home and follow social distancing measures. A medical education curriculum should be developed to avoid disruptions from pandemics and provide students with opportunities for continuous learning [7]. Furthermore, medical schools and other institutions that offer professional health education must modify their curricula and instruction techniques by utilizing cutting-edge distance-learning strategies, such as extended reality technology, e-learning resources, and simulation facilities, to address the COVID-19 pandemic.

Nursing is crucial in guaranteeing the excellence of healthcare services. The provision of services must conform to the ethical principles of nursing and the hierarchical progression of proficiency in the field. Nurses must acquire competencies such as knowledge, skills, attitudes, and abilities to efficiently perform their job and provide patients with high-quality care [8]. Professional nurses generally bear the burden of making clinical decisions. This study depicts the fundamental function of a nurse in delivering clinical services, which is a routine procedure for such professionals, to evaluate the quality of healthcare provided to their patients [9].

The continuous process of competency assessment aims to examine, monitor, and improve the skills and abilities of healthcare workers. Competencies can be classified under professional and clinical domains [10]. Professional competency includes adherence to ethical norms, comprehensive knowledge in healthcare legislation, effective decision-making skills, proficiency in professional development, and capacity to communicate efficiently [11]. Therefore, their education; training; experience; professional development; communication, clinical judgment, and decision-making abilities; and clinical competence contribute to nurses’ overall competence and clinical decision making (CDM) [12]. Furthermore, many studies found that factors such as age, ease with which one can take time off from work, workspaces with a clear vision, and strong interpersonal interactions can exert a substantial impact on fundamental nursing competencies [13].

Clinical decision making is a cognitive process concerned with problem recognition through the identification of cues or of relevant clinical features, data gathering, assimilation, analysis, evaluation and choice to produce an operational decision [14, 15]. From the findings of previous studies, the literature determined that CDM among nurses can be predicted by their possession of a nursing degree, as well as the number of hours worked. The findings of Saleh et al. [16], who observed that nurses’ level of education has a considerable favorable influence on their CDM ability, provided support to such findings. The factor that can account for the highest variability in CDM ability is professional occupational orientation, followed by the level of appointment, the area of clinical specialization, and age. Furthermore, Al-Maaitah, Shokeh [17]. determined that CDM is facilitated by commitment, authority, and autonomy, as well as ongoing supervision and feedback and effective communication. Meanwhile, the patient-nurse ratio, poor resource management, the healthcare system structure and culture, and lack of self-confidence, professional development, and knowledge are factors that decreased CDM practice among the study participants.

Thus, medical and nursing schools should use best practices to dynamically shift the focus on integrated learning and assessment based on knowledge obtained from previous pandemics. In addition, medical and nurse educators should develop useful rules or safeguards for providing clinical instruction, without compromising their safety and well-being, as well as the standards of medical education [18]. Synchronous and asynchronous online distance-learning strategies were found to have a similar effect on the critical-thinking and CDM abilities of first-year nursing students. Further research and critical discourse based on previous findings can be directed toward the development of procedures that will raise the standards of CDM and thinking. The findings of previous studies should be evaluated, and remote-learning techniques should be determined and implemented effectively [19].

This study focuses on identifying the predictors of and confounding factors that may affect CDM among nurses to provide empirical support to healthcare leaders. How such factors can affect CDM has yet to be examined; thus, increased research is necessary to fill the gap in the literature.

### Objectives

The main objective of this study is to determine the confounding factors that may affect the CDM of undergraduate nursing and midwifery students.

## Methods

### Study design and participants

This study used a cross-sectional design and was conducted on undergraduate nursing and midwifery students in Jordan, who were above the age of 18 years and enrolled in an online clinical training course during the COVID-19 pandemic. Data were collected from November to the end of December 2022.

The nursing students were identified with the help of the registration office. In this study, inclusion criteria sampling was used in the purposive sampling and snowball sampling was used because we intentionally selecting participants based on their characteristics, knowledge, experiences, or some other criteria as mentioned in the research objective and in the inclusion criteria.we recruit nursing and midwifery students who had successfully completed an adult healthcare course and who were residing in Irbid territory in Northern Jordan. The inclusion criteria for the participants were as follows: [1] must be at least 18 years old [2], must be currently enrolled in a nursing or midwifery program, and [3] must provide online informed consent and willingly participate in the study. The exclusion criteria were as follows: [1] individuals who were on leave for a period of at least one month and [2] students who were unreachable.

The sample size was determined using the following formula:

$$N = \lambda \frac{{(1 - {R^2})}}{R} + U + 1\,\left( {Cohen,\,1988} \right),$$

Where N is the sample size, λ is the effect size index, R^2^ is the proposed effect size, and U is the number of predictors.

$$N = 19\frac{{(1 - 0.13)}}{{0.13}} + 6 + 1 = 134.$$

### Data collection instruments

The data were collected using Google Forms, and the data extraction and recording were performed using Excel. The participants were recruited via social media and e-mail, specifically, the questionnaire link was sent to the participants through platforms such as Facebook and WhatsApp.

The initial component of the gathered information was the respondents’ sociodemographic data, namely, their age, gender, cumulative grade point average (CGPA), marital status, academic program, and duration of social media usage.

The second part of the questionnaire, which was the nursing decision-making instrument developed by Lauri (2002) ^[17]^, consisted of 24 items covering one domain. The response scale was a Likert scale ranging from 1 to 5, as follows: 1 - never, 2 - rarely, 3 - not rarely or not often, 4 - often, and 5 - nearly always or always. The questionnaire required around 4 min to complete. The questionnaire served as a valid and reliable instrument for evaluating CDM. The results of the exploratory factor analysis accounted for 69.07% of the variance, and the Cronbach’s alpha value was 0.92, which indicated a satisfactory level of internal Consistency [20].

Each respondent described how they made decisions in different patient care situations based on certain statements. The Nursing Decision-making Instrument-Revised was utilized as part of the online questionnaire.

### Human ethics and consent to participate declarations

Studies involving human participants are reviewed and approved by the institutional review board of Al-Balqa Applied University, which granted ethical approval for this study (26/3/2/790). The participants gave their informed consent electronically, rather than in writing. The justification for the lack of written informed consent was that the researchers clarified at the outset of the survey that participation in the study would be contingent on the participants’ willingness to complete the survey, which constituted their written informed consent. No in-person interviews were conducted; instead, a survey questionnaire was designed using Google Forms. The age requirement for participation in the study was 18 years or older; thus, neither a legal guardian nor a next of kin was required to provide informed consent. The researchers provided a clear explanation of the study’s objectives and anticipated outcomes. The researchers also informed the participants that their involvement was voluntary, and they could decline or withdraw from the study at any time without needing to provide a reason or facing any consequences. Moreover, the researchers assured the participants that their answers would be confidential.

### Data analysis

The procedure for analyzing the data was carried out with the help of Statistical Package for Social Sciences (version 26) for Windows. There are no missing data or outlayers despite the fact that the data was verified and cleansed.

In order to classify and evaluate the demographic data depending on the measurement level, descriptive statistics were utilized. These statistics included the mean, the standard deviation, and the frequency. The prospective determinants of the CDM of nursing and midwifery students were identified by the utilization of inferential statistics, namely through the utilization of basic and simple linear regression.

## Results

This study aimed to identify the factors that may influence the CDM of Jordanian nursing and midwifery undergraduate students after the COVID-19 pandemic. Factors such as years of study, age, daily social media usage in hours, CGPA, and enrollment in a nursing program had an impact on CDM.

A total of 269 nursing and midwifery students who matched the inclusion criteria were invited to participate in the study. However, only 224 students completed the survey, and 25 withdrew for a variety of reasons. Approximately 73% of the participants were female, with a mean age of 21 years.

Descriptive analysis was conducted to analyze the demographic data, and the findings are displayed in Table 1. Moreover, the majority (94.6%) of the participants was single, and 74.1% were enrolled in a nursing degree program. Over two thirds of the diploma nursing and midwifery student respondents received satisfactory or excellent grades and had a mean CGPA of 70.41. Furthermore, the majority of the students spent more than 6 h each day on social media, with a mean duration of 6.67 h.

**Table 1:** Demographic data

Variable	Frequency (%)
**Gender**	
Female	163 (72.8)
Male	61 (20.2)
**Program**	
General Nursing	166 (74.1)
Midwifery	58 (25.9)
**Marital status**	
Single	212 (94.6)
Married	12 (5.4)
**Variable**	**Mean**
Age	20.79
CGPA	70.41
Duration of social media usage in hours	6.67

**Table 2:** Simple linear regression analysis of predictors of nursing CDM

Variable	β (95% CI)	*P* value
**Gender (female)** Male	−0.408 (− 0.748, − 0.068)	0.019
**Duration of social media usage in hours**	−0.085 (− 0.112, − 0.057)	< 0.001
**CGPA**	−0.006 (− 0.016, 0.003)	0.193
**Age**	−0.008 (− 0.037, 0.021)	0.605
**Program** Nursing	0.110 (− 0.106, 0.325)	0.315
**Marital status (single)** Married	−0.075 (− 0.248, 0.097)	0.389

The researchers conducted simple linear regression to examine the link between a number of separate sociodemographic factors and predictor variables and how the nursing and midwifery students made clinical decisions. Simple linear regression was conducted through correlation analysis to identify the primary predictors of the CDM of the Jordanian students. Table 2 shows that gender and duration of social media usage in hours were the main confounding factors that affected nursing CDM. Being a male nursing student was found to be substantially correlated to poor CDM scores β = −0.408; *p* = 0.019). Moreover, social media use was significantly correlated to CDM among the Jordanian nursing and midwifery students. An increase of 1 h in the duration of social media use significantly decreased the mean nursing CDM score by 85 points. However, CGPA, age, enrollment in a midwifery program, and marital status were not predictors of nursing CDM, owing to their insignificant *p* values.

## Discussion

Nursing CDM is a complex process influenced by various factors. This study aims to fill the important gaps in the literature on nurses’ CDM. This study generally tries to identify the predictors of and factors that can influence CDM among nursing and midwifery students.

In contrast to other studies of a similar nature, which focused on how to enhance decision making, this study identifies the confounding factors that can affect CDM after the COVID-19 pandemic [21–23]. By utilizing a nursing and midwifery CDM questionnaire, this study identifies the predictors of CDM.

The most important finding of this study is that the students who received online clinical training have a lack of clinical experience. The students would be unable to adequately demonstrate their competence to work as hospital nurses. From the authors point of view; a possible explanation for this finding is that the nursing and midwifery students were confused by the unexpected commencement of online learning because of the COVID-19 pandemic and their disturbed lifestyles. The results shed light on the complex interplay between the students’ learning methods and the clinical learning environment [24].

Moreover, our investigation identifies two main variables that can affect nurses’ CDM. First, the results show that the more the social media usage, the lower the mean CDM score. Similarly, the students who use smartphones frequently at the clinic are likely to engage in cyberstalking behavior and have fewer professional decision-making skills compared with the other students [25]. Although the use of social media in the university setting has become increasingly prevalent [26], much remains to be understood about its effect on CDM among nursing and midwifery students. While nursing and midwifery students acknowledged utilizing personal mobile devices to access social media for learning purposes, the extent to which this usage impacts their competency development management (CDM) and its relevance to social media usage remain uncertain [27]. Additional investigation is required to explore the correlation between the utilization of social media by nursing and midwifery students and institutional and CDM outcomes. Although social media may offer advantages for nursing and midwifery students, such as facilitating professional collaboration and communication and promoting the dissemination of evidence-based information, its implementation in academic environments carries certain risks, including infringements on privacy and boundaries [28]. Furthermore, notwithstanding the initial favorable effects of social media, the majority of healthcare facilities have been sluggish to acknowledge its existence and incorporate it into their operations [29]. Nevertheless, it is probable that the utilization of social media and mobile devices in the healthcare sector will persist, as evidenced by the implementation of such tools by registered nurses in order to deliver innovative care and inspire favorable transformations in healthcare provision [30]. The capacity of social media to furnish nursing and midwifery students with immediate access to up-to-date evidence can aid in the development of their clinical reasoning and appropriate critical decision making (CDM), potentially leading to more timely care delivery and enhanced patient outcomes [31]. However, the use of personal social media by nursing students during class time should be acknowledged, and the potential dangers associated with its use in teaching laboratories should be discussed.

A comparison study conducted on nursing and midwifery student in Iran using paper based and electronic nursing process to assess their clinical decision making skill, the results showed that the implementation of an electronic nursing process increased the ability of student to take a clinical decision making in clinical setting and it was a safer method for providing the care of Patient [32].

One noteworthy finding of this study is that being male is significantly associated with low CDM score. Several possible explanations for this result may be considered. The nursing and midwifery profession remains female dominated [33]. Male nursing students in Muslim countries tend to feel trapped in nursing care and thus may have a low CDM threshold, because nursing is generally not regarded favorably as a profession by men [34, 35]. Working in the gynecology, obstetrics, maternity, and pediatric wards is generally regarded as unsuitable for male nurses [36]. By contrast, many female nurses reported having fewer nursing skills than their male counterparts. In CDM, gender plays a crucial role in nursing skills, competency, and the management of various procedures [37].

Nursing and midwifery students must develop their decision-making skills to meet patients’ demands in the dynamic healthcare environment.

The findings suggest that nurse educators, academicians, and managers work together to address or mitigate the factors that can hinder CDM practices and promote the adoption and use of factors that can enhance such practices. In addition, ongoing supervision and feedback along with continuous in-service training, can aid in expanding nurses’ knowledge base.

## Conclusions

The confounding factors that can affect the CDM of nurses and midwifery student have been a longstanding problem in universities and a critical issue that must be addressed, because they can lead to poor CDM. CDM process are crucial for nurses and midwifery students to be able to determine the best course of action for their patients. However, several factors can affect nursing and midwifery student ability to make effective decisions, such as their gender and social media usage. Thus, clinical instructors and faculty members must be aware of the potential risks and challenges associated with social media use for CDM. The identification of such confounding factors can help improve the quality of CDM among nursing and midwifery students. In other words, such students must be able to analyze and combine information, exercise discernment, and effectively use their choices to address patients’ issues within the framework of a diverse team.

### Research limitations

The use of a self-reported questionnaire can introduce bias into the examined relationships. The findings of this study, which was conducted in the Irbid region, may not be representative of CDM usage across Jordan. Thus, additional research is necessary to enhance the reliability of the findings and validate the research.

## Data Availability

The data supporting the findings of this study are available from the corresponding author, R. A., upon reasonable request.

## Abbreviations

CDM: Clinical decision making
